# A 16S rDNA PCR-based theoretical to actual delta approach on culturable mock communities revealed severe losses of diversity information

**DOI:** 10.1186/s12866-019-1446-2

**Published:** 2019-04-08

**Authors:** Hellen Ribeiro Martins dos Santos, Caio Suzart Argolo, Ronaldo Costa Argôlo-Filho, Leandro Lopes Loguercio

**Affiliations:** Department of Biological Sciences (DCB), State University of Santa Cruz (UESC), Pav. Jorge Amado, Rod. BR 415, Km 16, Salobrinho, Ilhéus, BA 45662-000 Brazil

**Keywords:** Microbial ecology and diversity, Community structure, Plant-associated bacteria, 16S rDNA metagenomics, Fingerprinting methods, PCR-RFLP, ARDRA, CAPS, Chimeric sequences, Hypervariable regions

## Abstract

**Background:**

Subunits of ribosomal RNA genes (rDNAs) characterized by PCR-based protocols have been the proxy for studies in microbial taxonomy, phylogenetics, evolution and ecology. However, relevant factors have shown to interfere in the experimental outputs in a variety of systems. In this work, a ‘theoretical’ to ‘actual’ delta approach was applied to data on culturable mock bacterial communities (MBCs) to study the levels of losses in operational taxonomic units (OTUs) detectability. Computational and lab-bench strategies based on 16S rDNA amplification by 799F and U1492R primers were employed, using a fingerprinting method with highly improved detectability of fragments as a case-study tool. MBCs were of two major types: in silico MBCs, assembled with database-retrieved sequences, and in vitro MBCs, with *Alu*I digestions of PCR data generated from culturable endophytes isolated from cacao trees.

**Results:**

Interfering factors for the 16 s rDNA amplifications, such as the type of template, direct and nested PCR, proportion of chloroplast DNA from a tropical plant source (*Virola officinalis*), and biased-amplification by the primers resulted in altered bacterial 16S rDNA amplification, both on MBCs and *V. officinalis* leaf-extracted DNA. For the theoretical data, the maximum number of fragments for in silico and in vitro cuts were not significantly different from each other. Primers’ preferences for certain sequences were detected, depending on the MBCs’ composition prior to PCR. The results indicated overall losses from 2.3 up to 8.2 times in the number of OTUs detected from actual *Alu*I digestions of MBCs when compared to in silico and in vitro theoretical data.

**Conclusions:**

Due to all those effects, the final amplification profile of the bacterial community assembled was remarkably simplified when compared to the expected number of detectable fragments known to be present in the MBC. From these findings, the scope of hypotheses generation and conclusions from experiments based on PCR amplifications of bacterial communities was discussed.

**Electronic supplementary material:**

The online version of this article (10.1186/s12866-019-1446-2) contains supplementary material, which is available to authorized users.

## Background

Studies on microbial diversity have long counted on classical methods of isolating microorganisms in vitro [1, 2]. This culturable diversity has also enabled bioprospection for new microbes and functions [3] with relevant applications in agriculture, health, industry and environment [4, 5]. However, as only a small fraction of microorganisms in nature (from 0.1 to 2% of the total) is culturable, molecular-based culture-independent methods have allowed a further understanding of microbial diversity [2, 6–8] by generating a large amount of taxonomic, phylogenetic and functional information, both for open environments and host systems [9–15].

The development of a last century’s breakthrough technique, the ‘PCR’ method (patent US4683195) [16], provided a great boost for the molecular methods, as target microbial DNA sequences could be amplified from heterogeneous mixtures of hosts’ or environmental samples. The vast majority of methods for microdiversity studies are based on PCR of ribosomal subunits genes. ‘Genetic fingerprinting’ is a first group of these techniques, including CAPS [17], ARDRA [18], T-RFLP [19], D/TGGE [20], A/RISA [7], and others [8, 15]. Although these methods allow for sequencing of operational taxonomic units (OTUs), their major purpose is to address the effects of different treatments on structure/complexity of communities and on population dynamics [15, 21]. Richness and relative abundance can be assessed, with a detection/counting range at the order of 10^1^ to 10^3^ OTUs per sample [10, 12, 17, 22]. A second group of methods employs high throughput sequencing (HTS) of PCR-amplified rDNA subunits [22–27]. These techniques have largely expanded OTU data generation by several orders of magnitude, allowing simultaneous analysis of richness, abundance and composition, and detection of much finer changes in the communities [8].

PCR shows widely acknowledged advantages, such as operational consistency, wide scope of investigation, excellent data generation / time spent ratios, and favorable costs and logistics for research [28, 29]. However, PCR also displays technical aspects prone to undesirable experimental variability, which greatly interfere quali- and quantitatively in the results from different biological systems [25, 30–32]. The type of lysis in DNA extraction, contaminating compounds, primers specificity, multi-template amounts, interference of non-target DNA, preferential amplification of certain sequences, and artifacts/chimera formation in the PCR are some of these limitations [25, 30, 33–35]. Countless studies have applied different 16S rRNA gene (rDNA) PCR-based methodologies to assess bacterial samples variation. However, the results often found for rarefaction curves (OTUs’ richness as a function of sequences’ abundance) suggest that the totality of diversity could not be accessed by the sampling method/effort employed. Interestingly, such conclusion has been obtained from either low-throughput fingerprinting methods or massive HTS data, despite their very large differences in the detectable number of OTUs [14, 15, 25, 32, 36–40]. Thus, a significant question remains: will it be possible to establish PCR-based conditions that would allow us to properly reach the entire collection of microbes existing in an environmental sample?

In addition to intrinsic biological differences, we should be aware of a possibly overwhelming variation among PCR-based studies [11, 32, 39], due to high interference of various technical factors [25, 30, 31, 40–43]. A simple approach to quantify losses in OTU’s detectability for 16S rDNA PCR-based methods, which directly compare theoretical maximum of bacteria present in a sample and what is actually identifiable after PCR from the community’s DNA, is convenient. In these circumstances, considering the well-reported intra-genomic variation found in several bacterial species [44–46], the use of a previously known number of culturable isolates to provide the theoretical number of OTUs present in a sample seems advantageous.

In this study, our objectives were two-fold. First, we aimed to evaluate the impact of the following factors on the outputs of diversity-assessments experiments: *(i)* presence of chloroplast 16S rDNA sequences from a tropical-tree source, *(ii)* number of amplification reactions, *(iii)* template composition, and *(iv)* preferential amplification of sequences in a complex DNA sample. Second, we assessed the differences (delta) in OTUs’ richness between the ‘theoretical’ in silico [47] and in vitro maximums, and the ‘actual’ values obtained in vitro, based on mock bacterial communities (MBCs) [24, 31, 32, 48, 49] assembled both with database sequences, and with DNA from culturable isolates. As a simple tool for this case study, a high-resolution PCR-RFLP-type method previously developed [50], based on polyacrylamide gradient-gel electrophoresis, was employed. The 799F and U1492R primers used in the experiments are regarded as an efficient pair to allow exclusion of chloroplast 16S rDNA amplification, or its separation from the bacterial amplicons in plant samples [2, 42, 51].

## Methods

### Plant sources of Bacteria and DNA

The 72 unique endophytic culturable bacterial isolates used in this work (see further below) belong to the Agroindustry Applied Microbiology Laboratory of the State University of Santa Cruz (LABMA-UESC, Ilhéus-BA, Brazil), and were previously isolated from pulp adhered to seeds from cacao fruits (*Theobroma cacao* L.) [52]. The isolates were purified by streaking at least three times until homogeneous colonies were obtained. To provide a tropical source for previously uncharacterized chloroplast and endophytic bacterial sequences, total DNA from leaves of an Atlantic Forest endemic tree, *Virola officinalis* Warb., were obtained from plants located in a same environment of 12.5 Km^2^, in the municipality of Belmonte-BA (S 15°17′, W 39°14′), at Southeastern Bahia (Brazil). The total leaf DNA from *V. officinalis* was extracted based on the Doyle and Doyle [47] method.

### Bacterial DNA extraction

The same extraction method of Doyle and Doyle [47] was used with modifications. For the endophytic bacteria isolated from cacao fruits (see above), an aliquot of 20 μL of each bacterial pre-culture was transferred to a 2-mL microtube with 1 mL of Terrific Broth (tryptone 1.2%, yeast extract 2.4%, glycerol 0.5%, KH_2_PO_4_ 0.17%, K_2_HPO_4_ 0.72%, in sterile water) and incubated at 28 °C for 48 h at 125 rpm. The cultures were centrifuged at 11,500 x *g* for 5 min, supernatant (SN) was discarded and 400 μL of extraction buffer were added, containing 1% CTAB, 0.2% β-mercaptoethanol, 2% PVP and the other components at the recommended concentrations [47]; in this buffer, Proteinase K and PVPP were not included, but 2% SDS was added. The samples were vortexed for 5 s and incubated at 65 °C for 30 min, with manual inversion of tubes every 10 min. At room temperature, 200 μL of 3 M KOAc were added, followed by hand shaking for 30 s and centrifugation at 10,000 x *g* (this same speed was used downstream) for 5 min. The SNs were transferred to 2-mL microtubes with 500 μL of chloroform: isoamyl alcohol (24:1; *v*/v), vortexed for 5 s, centrifuged for 10 min, and again removed to new 2-mL microtubes. For DNA precipitation, 125 μL of 10 M NH_4_OAc and 375 μL of isopropanol were added; samples were gently shaken, allowed to sit at − 20 °C for 1 h, and centrifuged for 15 min, with the SNs discarded. The pellets were washed twice by 200 μl of 70% ethanol and 5-min centrifugation, air-dried at room temperature for 50 min, resuspended in 40 μl of TE-RNase (10 μg ml^− 1^) and incubated at 37 °C for 40 min. Proper DNA quality for downstream procedures was checked by 1% (*w*/*v*) agarose gel electrophoresis in TBE buffer. Extracted DNA was quantified either by visual comparison with standards run in electrophoreses, or by NanoDrop ND-1000 droplet spectrophotometer (Thermo Scientific™).

### 16S rDNA amplification

The amplification of 16S rDNA was performed in two ways throughout the research. The first relates to PCR with universal primers 27F and 1492R, which spans nearly full-length of 16S rRNA gene [53], with an expected amplicon of ~ 1400 bp. The second relates to PCR with primers 799F and U1492R of the V5–V9 hypervariable region [2], with expected amplified fragments of ~ 700 bp. For this, we refer to ‘direct PCR’ when the 799F/U1492R primer-pair was used in the amplification procedure directly from extracted DNA as template (either from the endophytic bacterial isolates of cacao, or from total DNA from leaves of the *V. officinalis* tree; see above), and to ‘nested PCR’ when the product of a first 27F/1492R universal PCR was used as template for a second amplification with the nested primers 799F and U1492R.

PCR of the 16S rRNA gene with universal primers 27F (5′-AGAGTTTGATCCTGGCTCAG-3′) and 1492R (5′-TACGGYTACCTTGTTACGACTT-3′) [53] contained 2.5 μL of 10x Taq buffer, 1 μL of 50 mM MgCl_2_, 2.5 μL of 2 mM dNTPs, 0.2 μL of Platinum^®^ Taq Polymerase (5 U μL^− 1^, Invitrogen™), 5 pmoles of Primer 27F, 10 pmol of primer 1492R and 8 ng of DNA template, brought up to a final volume of 25 μL with ultra-pure water. The reactions were performed on GeneAmp PCR System 9700 (Applied Biosystems™) thermocycler under these conditions: 4 min at 96 °C, followed by 30 cycles of 30 s at 94 °C, 30 s at 57 °C and 1 min at 72 °C, and a final extension step at 72 °C for 10 min. Aliquots of 5 μL of each reaction were analyzed on 1% (*w*/*v*) agarose gel in TBE buffer.

For the primers 799F (5′-AACMGGATTAGATACCCKG-3′) and U1492R (5′-GGTTACCTTGTTACGACTT-3′) [2], ‘nested PCR’ contained 2.5 μL of 10X Taq buffer, 1.25 μL of 50 mM MgCl_2_, 2.5 μL of 2 mM dNTP, 0.2 μl of Platinum^®^ Taq Polymerase (5 U μL^− 1^) (Invitrogen™), 15 pmol of 799F, 7.5 pmol of U1492R, 0.25 μL BSA 0.1%, and 0.6 μL of the first 27F/1492R PCR amplification, in a final volume of 25 μL. For ‘direct PCR’ with the 799F/U1492R primers, always 8 ng of the extracted DNA were used as template per 25-μL reactions, which were performed in the same thermocycler model as follows: 3 min at 96 °C; 30 cycles of 20 s at 94 °C, 40 s at 58 °C and 40 s at 72 °C; a final extension at 72 °C for 10 min. The results were analyzed by agarose-gel electrophoresis as described. The use of the 799F primer supposedly improves the efficiency in separating the chloroplasts (~ 1,100 bp) and bacterial (~ 750 bp) 16S rDNA amplicons [2, 24, 25]. The bacterial 16S rDNA band was excised from gel and purified for sequencing and use in downstream procedures, using the PureLink^®^ Quick Gel Extraction Kit (Invitrogen™), following the manufacturer’s recommendations. The gel-purified DNA was quantified by NanoDrop ND-1000 (Thermo Scientific™).

### Sequencing of bacterial 16S rDNA amplicons

All kits, reagents, softwares and equipments described in this section were of Applied Biosystems™. A single expected-size 16S rDNA 799F/U1492R fragment was amplified from each cacao endophytic isolate, with direct sequencing of the gel-purified amplicons using the ABI-PRISM® 3100 Genetic Analyzer system. Sequencing reactions utilized 3 μL of the BigDye™ Terminator v3.1 Cycle Sequencing RR-100 reagent in a final volume of 10 μL, with DNA templates at ~ 50 ng, and 2.5 pmoles of the 799F primer, being performed in the GeneAmp^®^ PCR System 9700 thermocycler as follows: 3 min at 96 °C; 25 cycles of 10 s at 96 °C, 5 s at 55 °C and 4 min at 60 °C. The reaction products were precipitated with 75% isopropanol, washed with 60% ethanol, diluted in 10 μL of Hi-Fi formamide, denatured at 95 °C for 5 min, cooled on ice for 5 min and electro-injected in the automatic sequencer. The sequencing data were collected using the Data Collection v 1.0.1 program.

Approximate taxonomic identification of the sequences obtained from 16S rDNA of the endophytic bacterial isolates were achieved through the *BlastN* software (http://www.ncbi.nlm.nih.gov/BLAST/). For the purposes of this work, each bacterial isolate corresponded to an individual Operational Taxonomic Unit (OTU), whose single 799F/ U1492R amplicon was directly sequenced and returned a distinct accession number from the GenBank as the top hit, independently from the identity level (see Additional file 1: Table S1). This procedure was applied to a total of 96 culturable isolates from cacao fruits, having returned 72 unique accession numbers.

### Restriction analysis of 16S rDNA V5–V9 amplicons

16S rDNA-derived PCR products were amplified directly by primers 799F and U1492R either from individual DNA from each bacterial isolate (see above) or from pooled DNA from the in vitro ‘mock communities’ (see below). These products were digested with *Alu*I (AG/CT) restriction enzyme (Uniscience do Brasil) in reactions containing 0.8 μL of 10x enzyme buffer, 0.25 μL of the *Alu*I enzyme (10 U μL^− 1^), 2 μL of PCR reaction, brought up to final volume of 8-μL with ultra-pure water. The *Alu*I-digestion reactions were incubated in a water bath at 37 °C for 50 min, following enzyme manufacturer’s recommendations. For the amplified V5–V9 16S rDNA regions from the bacterial isolates, this 4-bp cutter was chosen because the discrimination power for the corresponding electrophoretic restriction profiles (see next) was convenient for downstream analyses.

For the separation of *Alu*I restriction fragments, a previously defined procedure with a high-resolution ability [50] was used. *Alu*I digestions were submitted to vertical electrophoresis in 5–11% polyacrylamide (*w*/*v*) gradient gel in 1x TAE buffer (20 mM Tris-acetate, 0.5 mM EDTA, pH 8) at 80 V for 16 h. Afterwards, the gels were stained for 30 min in the dark, using a solution composed of 15 μL of GelGreen™ for each 50 mL of distilled water (3: 10^4^ ratio), and photodocumented in Blue LED Transilluminator (Nippon Genetics Europe). The gel images were analyzed for fragments counting; this procedure allowed the unambiguous detection of individual fragments with a size-difference equal to, or greater than 5 bp.

Therefore, as a conceptual framework in this study, an OTU (see above) is not specifically related to a single restriction ‘fragment’, but rather to a restriction ‘profile’. In other words, a single culturable bacterial isolate (in vitro data) or an individual bacterial 16 s rDNA sequence (in silico data) retrieved from the database (does representing a single bacterial strain) were considered as an OTU.

### Mock bacterial communities (MBCs) with different proportions of chloroplast DNA

The main approach of this study was based on the composition of ‘mock bacterial communities’, or MBCs. The cpDNA used in this study was obtained from PCR amplification of total leaf DNA from the tropical *Virola officinalis* tree (see above), using the 799F and U1492R primers. Several leaf samples of this plant were used, providing the characteristic electrophoretic pattern of two bands [2, 51]. An expedited direct amplicon sequencing of the gel-purified ~ 1.1 Kb band confirmed this DNA to be from chloroplast (data not shown). These fragments were cut out from the gel and purified in sufficient amounts for downstream procedures by the same PureLink^®^ kit (Invitrogen™) indicated above.

Experiments on direct and nested amplifications with different proportions of PCR-amplified/purified cpDNA were done by adding different amounts of its DNA in 5- and 10-OTUs’ pooled-DNA MBCs. Treatments with cpDNA were 0% (control with only MBC DNA), 35, 65 and 100% (control with only cpDNA) of a 8-ng total DNA template in the reactions. Positive PCR control had template DNA from a single isolate. For nested PCR with ‘MBC + chloroplast DNA’, those percentages were established only for the first 27F/1492R amplification reaction. The amplification conditions with 799F and U1492R primers were the same described above; the results were analyzed after electrophoresis on 1% agarose gels stained with GelGreen™ and photodocumented in Blue LED Transilluminator.

### Theoretical and actual MBCs

MBCs were specifically assembled to quantitatively compare results between maximum ‘theoretical’ numbers of possible restriction fragments for the chosen enzyme (*Alu*I) and the ‘actual’ number of fragments obtained from in vitro experiments. The ‘theoretical’ maxima were based on counting differently sized *Alu*I-fragments per OTU, either in silico or in vitro, before assembling an MBC (‘pre-assembly’ data), whereas the ‘actual’ number of bands were counted for MBCs whose OTUs’ DNAs were pooled prior to PCR, digestion and separation (‘post-assembly’ data). Four different data sets of 16S rRNA genes from endophytic *Bacteria* were used for the MBCs: *(i)* 50 sequences of a wide variety of species obtained from the literature (Additional file 2: Table S2); *(ii)* 50 sequences reported for rice (*Oryza sativa*) [54]; *(iii)* 35 sequences reported for bean (*Phaseolus vulgaris*) [55]; *(iv)* 72 amplicons from 799F/U1492R PCR, each corresponding to a single endophytic bacterial isolate (OTU) from cacao fruits (see above). All these data sets were subjected to restriction analysis in specific manners, as described next.

#### In silico *Alu*I-digestion analysis of endophytic 16S rDNA sequences

We refer to this set as ‘pre-assembly’ *theoretical* data for in silico mock communities (items *(i)* to *(iii)* above). MBCs with increasing numbers of individual OTUs (sequences) were constructed based on the 50 entries indicated in Additional file 2: Table S2. The following stepwise procedure was performed using scripts developed in the PERL programming language (available upon request). First, the 799F primer annealing sites were identified for those 50 entries, to define the respective V5–V9 16S rDNA regions to be “digested” in silico. Second, each sequence was subjected to localization and counting of the respective *Alu*I restriction sites, with the numbers and sizes of generated fragments per sequence (*Alu*I–digestion profiles) being stored. Third, these 50 OTUs were subjected to 3000 rounds of randomization to compose groups of MBCs with 5, 10, 15, 20, 25, 30, 35, 40 and 45 OTUs; hence, each of these groups of ‘multiple-of-five OTUs’ had 3000 different MBCs, or replicates (with an obvious single MBC for the whole 50-OTUs’ group). With this procedure, 27,001 MBCs were generated (3000 replicates × 9 groups of multiple-of-five OTUs, plus one 50-OTUs’ MBC). For any given MBC, the total number of restriction fragments was the sum of those generated *individually* by each of its members, *prior* to the MBCs assembly (‘pre-assembly’ data). When fragments in a MBC were different by 5 bp or less, only one fragment was counted for that MBC (same resolution of the acrylamide-gradient gel). For the 50 rice and 35 bean sequences, the whole procedure was the same, so that the total number of in silico MBCs formed were also 27,001 for rice, and 18,001 for bean (3000 replicates × 6 groups of OTUs, plus 1 MBC with 35 sequences). For the graphical analyses, the average number of *Alu*I restriction fragments (dependent variable) for all the 3000 MBCs (replicates) in each multiple-of-five OTUs’ group (independent variable) was calculated and plotted.

#### In vitro *Alu*I analysis for individual amplicons of culturable bacteria

Similarly to the above, this data set also corresponds to *theoretical* ‘pre-assembly’ data for in vitro mock communities (item *(iv)* above). Bacterial DNAs extracted from the 72 cacao isolates (see Additional file 1: Table S1) were subjected to direct PCR with primers 799F and U1492R, followed by individual digestion of each amplicon with *Alu*I, and 5–11% polyacrylamide-gradient electrophoresis (see above). *Alu*I restriction profiles generated for each isolate were tabulated. The procedure was done twice for each isolate, with a third repetition for those in which the first two profiles came out not identical.

After collection and processing of the data from the individual OTUs (isolates), MBCs were then assembled in groups of ‘multiple-of-five’ OTUs, up to 30, similarly to the in silico procedure (see above). For each of these groups, five types of MBCs (five replicates per group) were composed with members from the 72 cacao isolates, based on the following criteria (see Additional file 1: Table S1 and Additional file 3: Table S3): ‘I’, complete randomization of OTUs; ‘II’, only non-*Bacillus* OTUs; ‘III’ and ‘IV’, 1/5 (20%) of *Bacillus* OTUs, with two distinct compositions (A and B); and ‘V’, only *Bacillus* OTUs (Additional file 3: Table S3). These criteria were established because the 16S rDNA V5–V9 amplicon-sequencing results of the 72 cacao endophytes indicated a high proportion of isolates most similar to the *Bacillus* genus (Additional file 3: Table S3). Importantly, for each of these five types of MBC, increasing number of OTUs was attained by stepwise addition of 5 extra isolates to the existing members in a multiple-of-five OTUs’ group, i.e., the members of the 5-OTUs’ group were included in the next 10-OTUs’ group, the members of this 10-OTUs’ group were included in the next 15-OTUs’ group, and so on. Hence, a total of 30 MBCs were established (5 MBC types × 6 multiple-of-five OTUs’ groups); since there was only 27 ‘non-*Bacillus*’ isolates in the 72-endophyte collection, the 30-OTUs’ MBC of this type (II) had to include three *Bacillus* to complete its composition (Additional file 1: Table S1 and Additional file 3: Table S3). For the graphical analyses, the number of ≥ 5 bp-different restriction fragments obtained for each of the 30 MBCs (dependent variable), was plotted for the corresponding multiple-of-five OTUs’ group (independent variable).

#### In vitro *Alu*I analysis for amplicons from pre-structured MBCs

This set of data corresponds to the *actual* ‘post-assembly’ data for mock communities. The same 30 MBCs described above (Additional file 3: Table S3) were now assembled prior to the PCR with 799F and U1492R primers, *Alu*I digestion and gradient-gel electrophoresis, by pooling extracted DNAs from each MBC member (isolate). The total amount of pooled DNA template was always 8 ng per 25-μL PCR, for any given MBC. All MBCs had equimolar amounts of DNA for all their members, independently from the number of OTUs. All procedures for PCR, enzyme digestion and electrophoresis were described above. The graphical analyses were performed as for the MBCs ‘pre-assembly’ data above, and the total number of restriction fragments for a given MBC was computed directly from the electrophoresis results.

#### Statistics

The data on number of *Alu*I fragments generated by the multiple-of-five OTUs were statistically analyzed by an unbalanced one-way ANOVA done as follows. The three in silico and the two in vitro (pre- and post-assembly) data sets were considered as five different ‘treatments’ (categorical independent variable). The ‘experimental units’ were the graphically-plotted average values of the ‘no. of bands per no. of OTUs’ (see Results), and the five means of each treatment were compared by the Tukey test (*p* < 0.05). Each treatment (data set) had a different number of experimental units (unbalanced no. replicates), which correspond to the number of multiple-of-five OTUs groups per set, i.e. 10 for Additional file 2: Table S2’s data, 10 for rice, 7 for beans, 6 for ‘pre-’ and 6 for ‘post-assembly’ in vitro data for MBCs (see Results). To verify normality of distribution for the values in these samples, the Lilliefors test (*p* < 0.05) was used. Statistical analyses were also performed to identify the best-fit type of regression (linear, exponential, logarithmic or geometrical curves) for all the five data sets described above, testing their significance by the *p* value. For all statistical procedures, the BioEstat 5.0 software was employed [56].

## Results

### Interference of chloroplast DNA and number of PCR amplifications

**Fig. 1:**
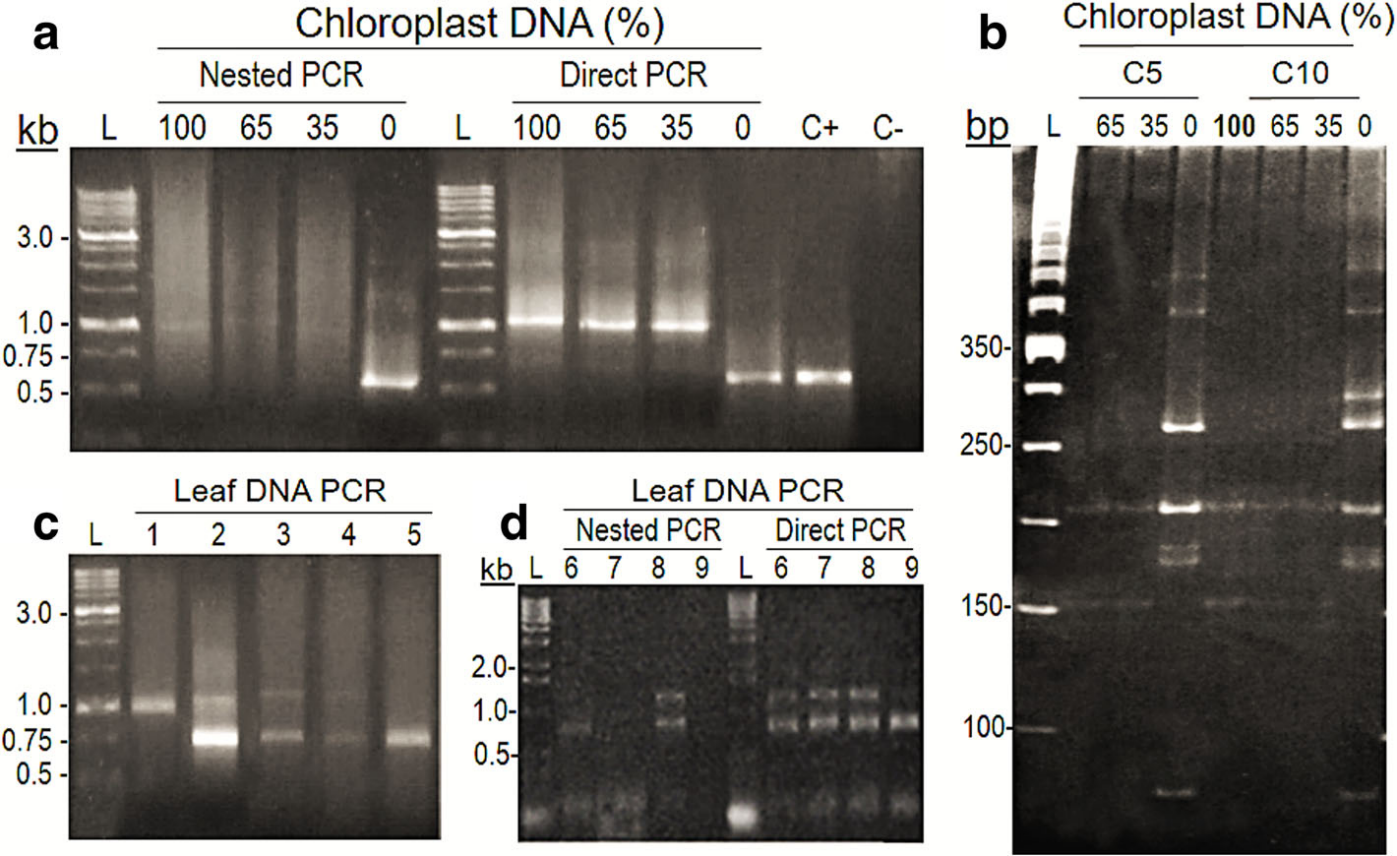
Effects of chloroplast DNA from *Virola officinalis* and number of amplification reactions on PCR products from the 16S rRNA gene amplified with primers 799F and U1492R. **a** Amplification patterns from ‘nested’ and ‘direct’ PCR done on pooled DNA from a 5-OTUs’ mock bacterial community (MBC), with different percentages of amplified/purified *V. officinalis* cp DNA (see Methods) added to samples prior to the PCR. Agarose and polyacrylamide gels were stained with GelGreen™. ‘L’: size ladder; ‘100’, ‘65’, ‘30’, ‘0’: percentage of cpDNA; ‘C+’: positive control (DNA of a single bacterial isolate); ‘C–’: negative control. **b** Chloroplast and bacterial 16S rDNA amplification patterns in *V. officinalis* total leaf-DNA samples from nested PCR; ‘1 to 5’: distinct plant individuals from the same area. **c** Patterns of nested and direct PCR from the same samples of total DNA extracted from *V. officinalis* leaves; ‘6 to 9’: distinct plants from same area. **d** *Alu*I restriction profiles from MBCs amplified with different percentages of cpDNA. ‘L’: size ladder (50 pb); ‘C5’: 5-OTUs’ MBC; ‘C10’: 10-OTUs’ MBC; ‘100’, ‘65’, ‘30’, ‘0’: percentages of cpDNA in the MBCs, prior to PCR, digestion and electrophoresis in 5–11% polyacrylamide gradient gel

The pattern of amplicons produced from the same samples were assessed by ‘Direct’ (1 PCR) and ‘nested’ (2 PCR) amplifications with the 799F and U1492R primers. Templates used were *(i)* pooled-DNA from the bacterial isolates (5- and 10-OTUs’ MBCs) mixed with previously uncharacterized PCR-amplified/purified cpDNA from the tree *Virola officinalis* Warb. (Myristicacaceae) (see Methods), and *(ii)* total leaf DNA from environmental samples of the same *V. officinalis* species (Fig. 1). The general pattern of amplicons was distinct between direct and nested PCR, although the presence of smears above and below the expected-size bands was similar for both cases (Fig. 1a). The amplification patterns were similar among all samples containing *V. officinalis* cpDNA (100, 65 and 35%) within nested or direct PCR. However, for nested PCR, a strong interference effect of the chloroplast sequences was observed, whereas for direct PCR, a preferential amplification over bacterial DNA was noticed instead; a clearer amplification of the bacterial 16 s rDNA was only achieved in the samples lacking cpDNA (Fig. 1a). As expected, the verified amplification preference for *V. officinalis* chloroplast DNA was reflected by the results of *Alu*I digestion (Fig. 1b), as the restriction patterns for the 100%-cpDNA control was the same as for MBCs with 65 and 35% cpDNA. Only when cpDNA was absent could the bacterial *Alu*I-digestion profile for the two communities be seen (Fig. 1b).

Similar interfering/confounding effects could be observed also for unknown communities from environmental samples, as in the case of *V. officinalis*-associated bacteria (Fig. 1c and d). Nested PCR on total-DNA samples extracted from the same organ (leaves), from individuals from the same sampling area (environment) showed varying amplification patterns of 16S rDNA: *(i)* only chloroplast amplification (upper band), *(ii)* varying intensities between chloroplast and bacterial amplicons (lower band), and *(iii)* only bacterial amplification were observed (Fig. 1c). Furthermore, differences between nested and direct PCR were also observed: while bacterial DNA was detected in all, and chloroplast in most samples by direct PCR, amplification failures were surprisingly found in some of the same samples by the nested PCR (Fig. 1d). For other leaf samples, however, there was an inverse pattern, with presence of bands in the nested and absence in the corresponding direct PCR (data not shown).

### Individual *Alu*I restriction analysis of culturable bacterial OTUs

**Fig. 2:**
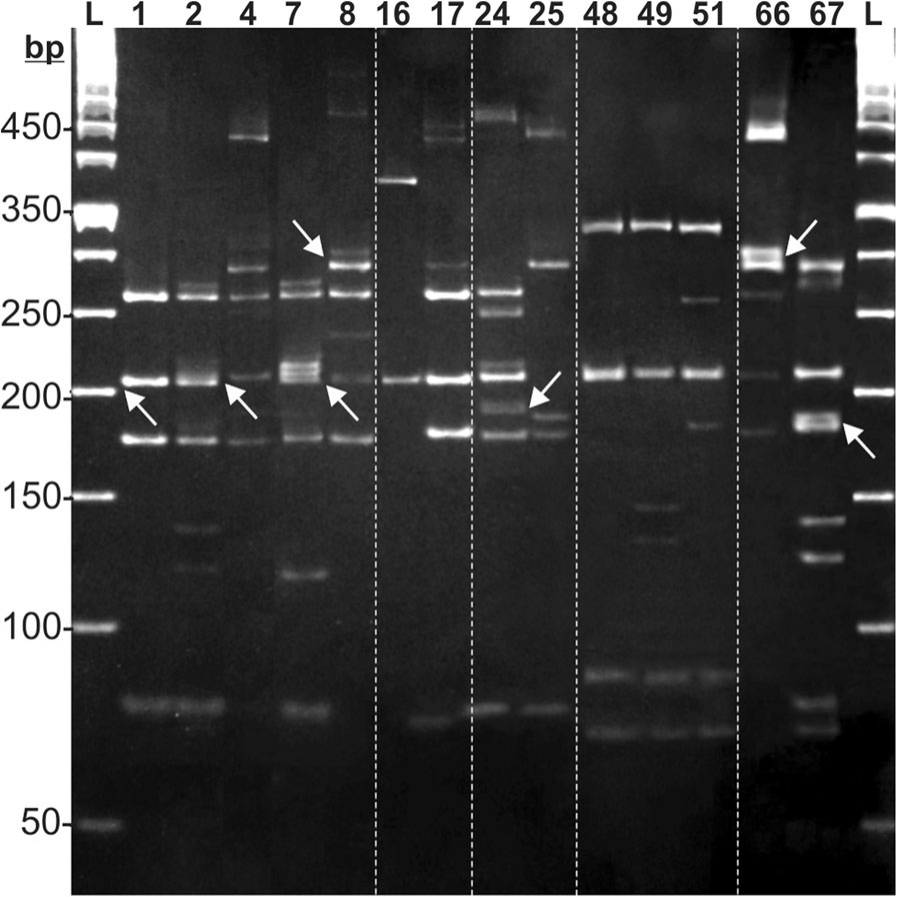
*Alu*I restriction profiles of bacterial endophytes (OTUs) from cacao, amplified by 799F and U1492R primers. OTUs were chosen to illustrate the variety of banding patterns and counts observed from individual *Alu*I digestions of the 72 culturable isolates. Amplicons from each OTU (lane) were digested by the 4-bp cutter restriction enzyme and electrophoresed in 5–11% polyacrylamide gradient gel. The ID numbers of isolates (Additional file 1: Table S1) appear at the top. Fragment sizes (in bp) are indicated on the left; ‘L’: 50-bp ladder. Arrows illustrate 5-bp size differences between fragments

A higher number of restriction fragments per OTU and a low resolution for fragments separation, thereby affecting the OTUs’ estimation in microbial communities, has been regarded as relevant limitations of PCR-RFLP (CAPS, ARDRA etc.) as a fingerprinting method [15, 21, 57]. To address this issue for the scope of this study, we have developed a very improved fragment-separation strategy, based upon 5–11% polyacrylamide gradient-gel electrophoresis [50], which showed to be very efficient in the range of 50 to 500 bp (Fig. 2). Only one frequent-cutter enzyme was used because simpler banding profiles related to possible polymorphisms for a single type of restriction site were more convenient for our purposes. As illustrated in Fig. 2 (arrows), this polyacrylamide gradient was able to distinguish bands with size differences as low as 5 bp. In a previous analysis with database-retrieved sequences, we found that an average of 3–5 *Alu*I bands could be expected per single V5–V9 region of 16S rDNA (data not shown). Considering that the whole amplification/digestion procedure was done at least twice for each isolate, it was noteworthy that some individual restriction profiles consistently displayed a higher number (6 to 11) of detectable fragments (Fig. 2). These *Alu*I-derived isolate-specific profiles were used in the mock-communities analyses (in vitro ‘pre-assembly’ MBCs data), as shown below. The significance and implications of the surprisingly large numbers of bands per OTU are discussed further.

### *Alu*I digestions of amplicons from mock bacterial communities (MBCs) assembled with increasing number of OTUs in vitro

**Fig. 3:**
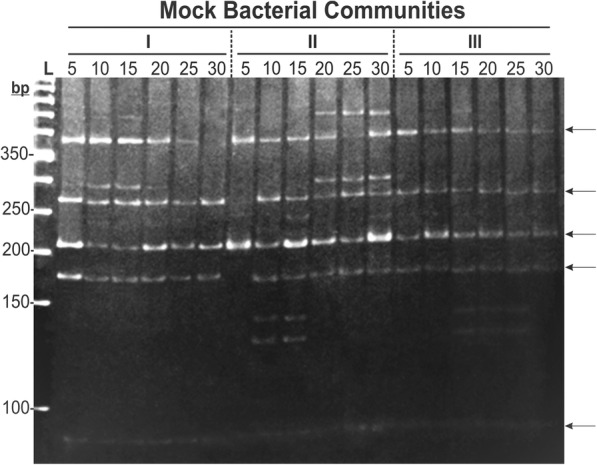
*Alu*I restriction profile of mock bacterial communities (MBCs) assembled with DNA from cacao bacterial isolates (OTUs) prior to PCR. The increasing numbers of OTUs per MBC are indicated on top of the respective lanes. ‘L’: size ladder (50 bp); I, II and III: types of MBCs composed according to criteria described in Additional file 3: Table S3. The increasing number of OTUs in each MBC type was established by stepwise addition of 5 new OTUs to the existing ones (Additional file 3: Table S3). Equimolar amounts of DNA extracted from each isolate were used to compose each MBC, prior to direct PCR with primers 799F and U1492R; the total amount of template DNA used in the PCR for each MBC was 8 ng. The whole volumes of *Alu*I digestions of the MBCs (8 μl) were applied on the 5–11% polyacrylamide gradient gels

This analysis aimed at verifying how the restriction patterns observable in MBCs assembled prior to PCR and digestion (‘post-assembly’ data) modify when the number of OTUs increases progressively. Our strategy was based on maintaining OTUs across MBCs by stepwise addition of 5 new OTUs to the existing ones (see Additional file 1: Table S1 and Additional file 3: Table S3). Surprisingly, the observed fingerprinting patterns indicated very little variation in number and position of bands (Fig. 3, arrows) among the three types of MBCs (I, II and III) and among MBCs with increasing numbers of OTUs, despite their distinct compositions. In other words, assuming complete digestion for all samples (see Methods), the total number of representative fragments for the communities (indicative of OTU richness) did not change, or increased very little with higher numbers of isolates in the MBCs. For example, in the MBCs from type II, additional bands in the community appeared between 5 and 15 OTUs, but only positional changes in the profiles was further observed above 20 OTUs; disappearance and emergence of bands were noticed, which roughly kept the same number of detectable bands among MBCs (Fig. 3). It is also interesting that, for the type I, the MBC with the lowest number of bands was the one with the highest number of OTUs (Fig. 3). Since the 5–11% acrylamide gradient allowed discerning fragments with ≥ 5-bp differences, an apparently higher band intensity for some of ~ 210-bp fragments (e.g. type-II MBC samples with 5, 15 and 30 OTUs, and type-III MBCs with 10 and 20 OTUs; Fig. 3) may have been due to two or more fragments different in less than 5 bp. As indicated in Additional file 3: Table S3, some of the OTUs illustrated in Fig. 2 were included in the MBCs, so that, not unexpectedly, we could spot same-size bands for both experiments (Figs. 2 and 3).

### Detectability losses from MBCs by ‘theoretical’ to ‘actual’ delta

**Fig. 4:**
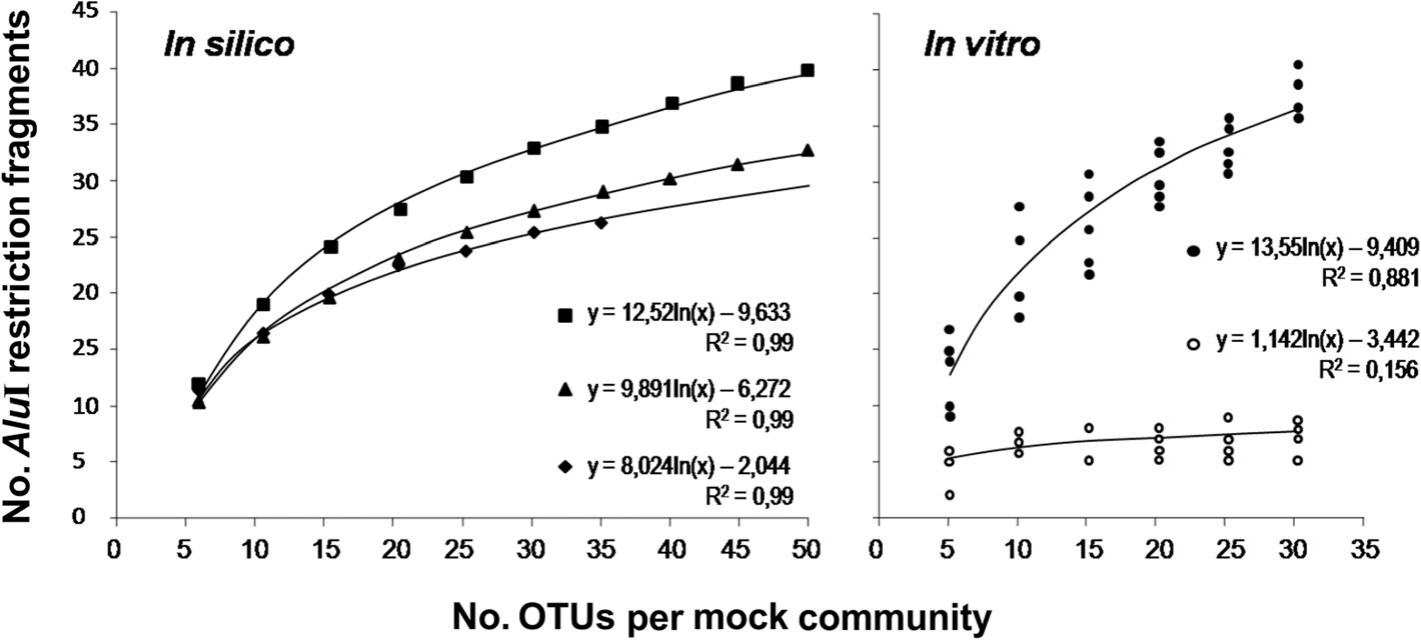
Theoretical and actual *Alu*I restriction fragments of mock bacterial communities (MBCs). For all cases, the MBCs were assembled with 5-OTUs’ increments, according to the graphs. For both in silico and in vitro analyses, restriction fragments equal to, or less than 5-bp difference were considered as a single band and counted only once per MBC (see Methods). In silico analysis: the number of *Alu*I fragments was defined from 16S rDNA sequences from various endophytic bacteria from database, followed by computational processing and restriction analysis by free online software (see Methods). ‘■’: sequences (OTUs) from the literature (Additional file 2: Table S2); ‘▲’: sequences from rice, *Oryza sativa* [54]; ‘♦’: sequences from bean, *Phaseolus vulgaris* [55]. The plotted data were the averages of maximum numbers of restriction fragments from 3000 random MBCs for the Additional file 2: Table S2’, rice’s and beans’ OTUs. In vitro analysis: the *Alu*I fragments of 16S rDNA amplified by 799F/U1492R were obtained in two ways: ‘●’: pre MBC assembly ‘theoretical’ data; DNA extracted from cacao bacterial isolates (Additional file 1: Table S1) were individually subjected to PCR / *Alu*I digestion (Fig. 2), computing the number of fragments per isolate prior to defining the MBCs; for each no. of OTUs on the horizontal axis, the total number of fragments for each of the 5 MBCs (Additional file 3: Table S3) were plotted. ‘○’: post MBC assembly ‘actual’ data; restriction fragments were obtained from PCR and digestion of previously pooled equimolar amounts of isolate DNAs, according to the no. of OTUs per MBC (Fig. 3). These experiments were repeated at least twice for all isolates and their MBCs. The respective regressions, equations and coefficients of determination are indicated in the graphs

The magnitude of information losses after direct PCR of 16S rDNA with primers 799F/U1492R, using MBCs with previously known numbers of OTUs and absence of cpDNA, was assessed. The approach was based on quantifying maximum (theoretical) values for the number of restriction fragments that individual OTUs could generate (‘pre-assembly’ MBC data), and verify the loss of fragment counts caused by PCR / *Alu*I digestion of previously-pooled DNAs of the same OTUs (see Fig. 3). To establish those theoretical maxima, the *Alu*I cuts were counted for each OTU separately, both in silico from database sequences (Additional file 2: Table S2; [58, 67]), and in vitro from individual 16S rDNA amplicons of the 72 cacao endophytes (Additional file 1: Table S1; Fig. 2). Following a regression analyses for the best-fit curve, the results expectedly indicated that the numbers of *Alu*I fragments per no. of OTUs in the MBCs increase logarithmically, tending towards a *plateau* (Fig. 4). R^2^ coefficients of ≥ 0.995 were found for the logarithmic regressions of the three in silico data sets (Fig. 4a), and of 0.881 for the ‘pre-assembly’ MBC data in vitro (Fig. 4b). The curves’ shape indicated that more *Alu*I fragments are yet to be found with higher numbers of OTUs in the MBCs.

For the ‘post-assembly’ (actual) MBC data, on the other hand, the best adjustment was found for a geometrical curve (R^2^ = 0.198) when compared with the logarithmic regression (R^2^ = 0.157), although both type of regressions were statistically significant (*p* < 0.05). The 50 bacterial sequences from the literature (Additional file 2: Table S2) showed the highest numbers of *Alu*I fragments with increasing numbers of OTUs (Fig. 4a), which was similar to that obtained from in vitro ‘pre-assembly’ MBC data for cacao isolates (regression coefficients of 12.52 and 13.55 respectively, Fig. 4a and b). For the rice and bean sequences, smaller regression coefficients (9.89 and 8.02, respectively) were found (Fig. 4a). The variation in the number of *Alu*I fragments per no. of OTUs was fairly homogeneous, with standard deviations varying from 0.78 to 2.25 restriction fragments for all plotted averages of the three in silico sets of 16S rDNA sequences (Fig. 4a).

Unbalanced one-way ANOVA was employed to test for statistical differences among the five sets of in silico and in vitro data (five treatments of a categorical independent variable), assuming each plotted average of ‘no. of *Alu*I bands’ per ‘no. of OTUs’ as an experimental unit (see Methods). The four overall means for the ‘theoretical’ in silico and in vitro data sets (treatments) were not significantly different from each other. However, a significant decrease was observed in the overall mean for the ‘actual’ treatment, with a remarkable loss of detectability in the number of restriction fragments for the same MBCs when comparing theoretical and actual data (Fig. 4b). The number of bands obtained from ‘pre-assembly’ (theoretical) MBCs was 2.3 to 8.2 times greater than those obtained from ‘post-assembly’ (actual) MBCs (Fig. 4). In addition, for the former, the maximum number of restriction fragments increases along with higher no. of OTUs in the MBCs, whereas for the latter, this number stabilizes for MBCs with as low as 10 OTUs (Figs. 3 and 4b). In fact, based on the shapes of the in vitro curves, it appears that the delta between theoretical and actual data is likely to become even larger if more OTUs are added to MBCs.

## Discussion

The use of HTS methods has drastically increased the outcomes in information, precision and reaches of research in microbial diversity. Yet, low-throughput methods remain useful when simple variation on profiles of detectable operational taxonomic units (OTUs) is a sufficient response variable [12, 13, 17, 59–65]. Independently of the analyses scale (fingerprinting or HTS), though, the uncertainty about the maximum possible number of accessible OTUs has been an issue, as rarefaction curves mostly suggest the sampling efforts are not enough to cover the totality of diversity [11, 12, 22, 30, 38, 54, 66, 67]. It is our contention that the possibility of reaching the totality of a given microdiversity is in fact hampered by informational losses or bias typical from the investigation routine. For 16S rDNA-based amplification methods, such a detectability issue is caused by a variety of factors related to the PCR [1, 27, 32, 41–43, 46, 54, 59, 62]. In this study, we evaluated the magnitude of losses in bacterial diversity accessible by PCR with 799F/U1492R universal primers, choosing ‘endophytes’ as a common ground to provide comparable in silico and in vitro samples. Our strategy was based on a ‘theoretical-minus-actual-data’ approach, in which culturable endophytic isolates [52] were used as individual OTUs to assemble mock bacterial communities (MBCs). While the ‘theoretical’ data were obtained from individual OTUs, the ‘actual’ data came from pooled DNA from these same OTUs prior to PCR. Since their 799F/U1492R-amplified regions were not cloned prior to sequencing, some lower-quality sequences yielded lower levels of identity with those from the *GenBank* (Additional file 1: Table S1). This was likely caused by non-specific primer binding and/or by intragenomic variations in 16S rDNAs, which have been shown to be less rare than commonly thought [45, 46, 68, 69] (see further discussion below). Nevertheless, despite such a limitation, this set of 72 unique culturable bacterial isolates (OTUs) allowed a proper composition of MBCs for the experiments (Figs. 2, 3 and 4).

Highly specific primers are essential to investigate bacterial diversity in environmental samples [42, 65], mainly in cases involving endophytes. Primer-pairs that include the 799F, covering the V5–V9 hypervariable region of 16S rDNA, have been suggested to either exclude amplification of cpDNA, or properly separate bacterial from chloroplast amplicons [2, 24, 25, 51, 54, 67]. The northeastern Brazil is a tropical region rich in unexplored biodiversity, as it harbors the largest remnants of the Atlantic rainforest, considered as ‘hotspots’ for conservation practices [70, 71]. *Virola officinalis* is an endemic tree from this region [72] that has not been previously studied with respect to its chloroplast features. Hence, cpDNA from *V. officinalis* likely has sufficient sequence homology with the 799F/U1492R primer-pair, as it interfered with bacterial amplification, showing preference for primer annealing in some cases (Fig. 1a and c). A similar result of cpDNA amplification with 799F has been reported [22]. It has to be acknowledged that, under high cpDNA interference, changes in the number of bacterial OTUs in a sample may be undetectable (Fig. 1b). The amount of chloroplasts in plants can vary markedly, depending on the species, cell type/position, age, physiological status, differential incidence of light, etc. [73]; amounts of cpDNA such as 10,000 copies per leaf cell can be found [74]. In fact, such a level of variability and potential interference of cpDNA in PCR (Fig. 1) may help to explain a significant part of the widely reported inter-tissues/inter-sites differences in microbial communities of the same plant [13, 25, 29, 39, 53]. Therefore, a very careful experimental planning is needed when addressing endophytic bacterial diversity; a relevant alternative is using extraction procedures able to isolate chloroplasts from the total extractable DNA [25, 73, 75, 76].

In addition to cpDNA interference, two other technical factors appeared to be an issue. The amount of template DNA we used is not unusual in bacterial diversity experiments [22, 77], so that the smear observed, especially above the expected-size amplicons, may be due to chimeric amplification [1, 41, 48, 78]. Depending on the biological/experimental system, unspecific priming in PCR leads to electrophoretic smears that may not be possibly eliminated. Also, the number of PCRs can interfere in the final amplification output [23, 41, 48], likely by interacting with the cpDNA and primers specificity (Fig. 1a and d). Taken together, these results suggest that research on microdiversity in tropical plants will require efforts to check for applicability and efficiency (on any low- or high-throughput platform) of 16S rDNA specific primers, adjusting experimental settings for more consistent, reproducible and broad amplification of associated bacteria [30, 35, 40, 43, 78]. This appears to be necessary, especially when dealing with previously under/unexplored plant species.

To obtain a theoretical maximum of *Alu*I restriction fragments closer to a practical reality, the culturable OTUs from cacao were assessed individually (examples illustrated on Fig. 2). In this regard, various aspects are worth discussing. First, the 5–11% polyacrylamide gradient gels [50] remarkably enhanced the resolution on the range of 50 to 500 bp, such that fragments different by at least 5 bp could be unequivocally counted. Second, the isolate-specific restriction patterns observed indicated that our strategy of considering only unique OTUs for the MBCs assembly, and so for the generation of the maximum theoretical number of bands, was appropriate. Third, it was interesting that the number of *Alu*I fragments obtained for several isolates was a lot higher than five, which is a maximum amount reasonably expected for a single 16S rDNA’s 799F/U1492R amplicon. Although non-specific and/or chimeric amplifications could explain additional restriction fragments, these seem to be a less likely explanation, since each of the 72 isolates generated a single and clear-cut expected-size PCR amplicon without smears or any non-specific amplification (data not shown). The presence of such extra *Alu*I bands could be alternatively explained by intragenomic heterogeneity [44–46, 79], i.e. the presence of more than one 16S rDNA sequence per cell, possibly formed by horizontal transfer/exchange [79–81]. The banding patterns of the *Bacillus*-like isolates (‘1’ to ‘25’ in Fig. 2; see Additional file 1: Table S1), with different number of fragments per OTU, but with various size similarity among OTUs, add support to this idea. Furthermore, the variable band intensities among isolates might be related to different copy numbers of the sequences [45, 46, 82]. This whole view is consistent with results from a survey in 224 *Bacillus cereus*-group strains that showed an average number of 6.5 16S rDNA operons per cell [83]. Since the cacao endophytes here reported [52] can be novel tropical strains/species, research is currently underway to provide in-depth characterization of these bacteria (to be published elsewhere).

The distinct *Alu*I profiles for the isolates were consistently reproducible between replicates and experiments (not shown). However, despite the progressively higher complexity in DNA templates led by more OTUs in the MBCs (Additional file 3: Table S3), *(i)* a reduced-number of fragments, *(ii)* a little variation in the banding profiles (with position similarity for most fragments), and *(iii)* an appearance and disappearance of bands were all observed among ‘actual’ MBCs (Fig. 3). These results contrasted to what would be more logically expected based on Fig. 2, i.e. an increase in detectable fragments along with higher numbers of OTUs. These pieces of evidence suggest a strong bias in the PCRs, in which the primers likely had a binding preference for specific sequences (OTUs) within the MBCs [40, 84, 85]. A similar phenomenon of preferential annealing (“sequestration”) of primers, altering the final observable structure of microbial communities, has been also observed on mock assemblies assessed by HTS platforms [39, 41]. Since the amount of template in the MBCs was always the same, made of equimolar amounts of DNA from participating isolates, our results also suggest that most abundant OTUs in a sample will not necessarily be amplified preferentially, as it has been long- and logically-assumed. This certainly has a significant impact on estimates of diversity indexes in natural communities [11, 62, 79, 85–87], independently of the analytical platform used.

It is possible that working with more than 30 OTUs in the MBCs could have yielded more bands, although this trend was not observed (Figs. 3 and 4b). Further experiments are warranted for an in-depth assessment of such a scale issue, as well as to test whether very low levels of template concentration (such as 0.27 ng per isolate as in the 30-OTUs MBCs) could interact with primer affinity to define the final amplification output of complex communities. It is important to highlight that only one restriction enzyme was used in this study to simplify the restriction profiles. The simultaneous use of other restriction enzyme(s) as in usual PCR-RFLP-type studies [9, 18, 88] would have increased the complexity of banding profiles, likely turning the data analysis into a cumbersome process; the fact that a higher number of *Alu*I fragments was found for many isolates individually (Fig. 2) proved that our single-enzyme approach was appropriate for our objectives. With the level of resolution attainable in this study with the 5–11% polyacrylamide-gradient gels (Figs. 2 and 3), a supposedly advantageous use of additional restriction enzymes has to be pondered for PCR-RFLP/CAPS/ARDRA types of analysis on environmental samples.

Based on our MBCs’ analyses (Fig. 4), the in vitro and in silico treatments defined a similar ‘theoretical’ maxima of restriction fragments for a known number of OTUs. In other words, five types (replicates) of MBCs for the in vitro ‘pre-assembly’ data (Additional file 3: Table S3) provided an output that was not statistically different than 3000 replicates of MBCs for the in silico data (Fig. 4). Considering the significant failure in fragment detection for ‘actual’ MBCs (Figs. 3 and 4), the loss of phylogenetic information from environmental samples of microorganisms seems to be very relevant for methods based on 16S rDNA PCR, independently from the community’s complexity [25, 32, 57, 78, 89]. In view of the various interfering factors here discussed, a direct experimental access to all possible microbes in a sample through PCR will likely be unfeasible, even for high-throughput techniques. Therefore, for the vast majority of studies, the current explanation given for rarefaction curves that tend to, but not reach a plateau, might need to be reconsidered: this likely happens not because the sampling effort is insufficient, but rather because the totality of a microdiversity simply cannot be reached by PCR-based methods. The direct access to microbial communities without relying on PCR, such as using a true metagenomics approach [31], may possibly be a feasible alternative to solve this whole issue.

## Conclusion

Here we reported a simple method to quantify the losses in microbial detectability in a biological sample, based upon in silico and culturable mock bacterial communities (MBCs), and upon the estimated differences between ‘theoretical’ and ‘actual’ number of OTUs. Our results indicated that, independently of the scale of the analysis, environmental samples of microorganisms subjected to universal-priming PCR can show a severely biased and misestimated number of OTUs. The extent of losses and misinformation can be remarkable, mostly due to preferential amplification for sub-sets of sequences in the sample, and/or varying levels of interference led by intragenomic variability. If dealing with endophytic communities, further interfering effects on primer-binding can be caused by cpDNA. These confounding aspects must not be overlooked in studies on microbial diversity, as they can alter the outputs of richness, abundance and composition of OTUs [1, 11, 24, 31]. It seems clear that true sources of variation among environmental microbial communities are not only the natural differences between samples, but also the intrinsic interfering effects of the research methodology. Despite the analytical power, depth and reach of high-throughput sequencing approaches, there are circumstances where simple observation of changes in robustly detectable OTUs will suffice for the research objectives, such as in multi-samples assessments of treatments effects on structure and dynamics of microbial communities [15, 21, 59]. Hence, the great improvement in OTUs’ characterization allowed by the 5–11% polyacrylamide-gradient electrophoresis [50] appeared as an interesting alternative, mainly for lab settings where infrastructure and/or logistics for high-throughput methods are routinely lacking [44, 61, 79, 87, 90]. For research designs relying upon PCR-based methods for microdiversity studies [46, 88], we hope this study has contributed to a greater awareness for the need of not only a comprehensive knowledge on the biological systems under study, but also a maximum control of intrinsic factors of variation, mainly those related to universal-primed PCR on 16S rDNA.

## Additional files

Additional file 1: **Table S1.** Approximate identification of culturable endophytic bacterial isolates from cacao, based on direct amplicon sequencing of the 16S rDNA V5–V9 hypervariable region. This table shows the overall level of identity with the top hit (accession number) retrieved by the *BlastN* search performed with the direct-amplicon sequences from each isolate used to compose the mock bacterial communities (MBCs). The likely reasons for those results are explained in the table’s footnote. (DOCX 32 kb)

Additional file 2: **Table S2.** Selected bacterial endophytes for in silico restriction analysis of V5–V9 region of 16S rDNA with *Alu*I enzyme. In this table, we present the results from a systematic search for a variety of rRNA gene sequences specifically from endophytic bacteria previously described, which were used for the theoretical in silico MBCs used in this study. For the chosen sequences shown in the Table, we aimed at covering the widest possible spectrum of bacterial species, isolated from all major plant organs. (DOCX 38 kb)

Additional file 3: **Table S3.** Composition of mock bacterial communities based on culturable endophytic isolates from cacao. In this table, one can see all the combinations of individual isolates used to compose the MBCs for the actual (post assembly, prior to PCR) communities, including all number of OTUs assessed (i.e., 5, 10, 15, 20, 25 and 30 OTUs). The information on this table is relevant for a better understanding of data shown in Figs. 3 and 4. (DOCX 88 kb)

## Abbreviations

(A)RISA: (Automated) ribosomal intergenic spacer analysis
(T-)RFLP: (Terminal) restriction fragment length polymorphism
ARDRA: Amplified ribosomal DNA restriction analysis
CAPS: Cleaved amplified polymorphic sequences
cpDNA: chloroplast DNA
CTAB: Cetyl trimethylammonium bromide
D/TGGE: Denaturing and temperature gradient gel electrophoresis
EDTA: Ethylenediamine tetraacetic acid
HTS: High throughput sequencing
MBC: Mock bacterial communitie
OTU: Operational taxonomic unit
PCR: Polymerase chain reaction
PVP: Polyvinylpyrrolidone
PVPP: Polyvinylpolypyrrolidone
rDNA: ribosomal RNA gene
rRNA: ribosomal RNA
SDS: Sulfate dodecyl sodium
SN: Supernatant
TBE: Tris-Borat-EDTA
TE: Tris-EDTA
*V. officinalis*: *Virola officinalis*
