# Supplementary material for: A 16S rDNA PCR-based theoretical to actual delta approach on culturable mock communities revealed severe losses of diversity information
Source: BMC Microbiol. 2019 Apr 8;19:74. doi: 10.1186/s12866-019-1446-2 (PMC6454784; doi:10.1186/s12866-019-1446-2)
Supplement: Supplementary file 1 — Table S1. Approximate identification of culturable endophytic bacterial isolates from cacao, based on direct amplicon sequencing of the 16S rDNA V5–V9 hypervariable region. This table shows the overall level of identity with the top hit (accession number) retrieved by the BlastN search performed with the direct-amplicon sequences from each isolate used to compose the mock bacterial communities (MBCs). The likely reasons for those results are explained in the table’s footnote. (DOCX 32 kb) [file 12866_2019_1446_MOESM1_ESM.docx]

Supplementary Material

**A 16S rDNA PCR-Based Theoretical to Actual Delta Approach on Culturable Mock Communities Revealed Severe Losses of Diversity Information**

**Hellen Ribeiro Martins dos Santos, Caio Suzart Argolo, Ronaldo Costa Argôlo-Filho*, Leandro Lopes Loguercio.**

** Corresponding author:*

E-mail: ronaldoargolo@yahoo.com.br

Post-graduation Program in Genetics and Molecular Biology (PPG-GBM)

Dept. Biological Sciences (DCB)

State University of Santa Cruz (UESC)

Pav. Jorge Amado,

Rod. BR 415, Km 16, Salobrinho,

Ilhéus-BA, 45662-000

BRAZIL

**1. Supplementary Tables**

**Table S1**: Approximate identification of culturable endophytic bacterial isolates from cacao, based on direct amplicon sequencing of the 16S rDNA V5–V9 hypervariable region.

| **Isolate ID** | ***BlastN* results (16S rDNA)** | **Access number retrieved** | **identity (%)** |
| --- | --- | --- | --- |
| 1 | *Bacillus altitudinis* strain BT 98 | KJ848598.1 | 84 |
| 2 | *Bacillus altitudinis* strain JF2 | KC171985.1 | 79 |
| 3 | *Bacillus amyloliquefaciens* strain HD34 | KT368090.1 | 99 |
| 4 | *Bacillus atrophaeus* strain NBF1 | HQ256518.1 | 71 |
| 5 | *Bacillus cereus* LV11 | KU705859.1 | 100 |
| 6 | *Bacillus cereus* strain M1GT | KX033490.1 | 100 |
| 7 | *Bacillus firmus* | EF526504.1 | 70 |
| 8 | *Bacillus licheniformis* strain KYLS-CU01 | KF111800.1 | 82 |
| 9 | *Bacillus pumilus* | GQ861537.1 | 71 |
| 10 | *Bacillus pumilus* | KU922935.1 | 85 |
| 11 | *Bacillus pumilus* strain AUCAB16 | JN315777.1 | 99 |
| 12 | *Bacillus pumilus* strain C1C5502 | KR677555.1 | 80 |
| 13 | *Bacillus pumilus* strain D30 | KM488477.1 | 85 |
| 14 | *Bacillus pumilus* strain DL12 | JF694824.1 | 79 |
| 15 | *Bacillus pumilus* strain DSMZ27 | AY456263.1 | 85 |
| 16 | *Bacillus pumilus* strain IARI-SL-5 | JX645203.1 | 70 |
| 17 | *Bacillus pumilus* strain IHB B 12534 | KJ767390.1 | 99 |
| 18 | *Bacillus pumilus* strain L1 | KT937148.1 | 100 |
| 19 | *Bacillus pumilus* strain MW-1 | HM027879.1 | 79 |
| 20 | *Bacillus pumilus* strain p7_H06 | JQ831639.1 | 78 |
| 21 | *Bacillus pumilus* strain PJRB5 | KM066948.1 | 84 |
| 22 | *Bacillus pumilus* strain RCPS-5 | HM172502.1 | 85 |
| 23 | *Bacillus pumilus* strain S8-07 | EU620415.1 | 99 |
| 24 | *Bacillus pumilus* strain SH-B9 | CP011007.1 | 82 |
| 25 | *Bacillus pumilus* strain SQU P001 | KU220846.1 | 83 |
| 26 | *Bacillus pumilus* strain SW-3 | KC813157.1 | 77 |
| 27 | *Bacillus pumilus* strain TP-Snow-C22 | HQ327131.1 | 82 |
| 28 | *Bacillus pumilus* strain ZK1 | JQ773350.1 | 73 |
| 29 | *Bacillus safensis* strain AL-8 | HQ848126.1 | 84 |
| 30 | *Bacillus safensis* strain FFA38 | JN092820.1 | 80 |
| 31 | *Bacillus safensis* strain IHB B 14105 | KM817280.1 | 100 |
| 32 | *Bacillus safensis* strain MUGA141 | KJ672329.1 | 74 |
| 33 | *Bacillus* sp. 01082 | EU520309.1 | 81 |
| 34 | *Bacillus* sp. BAB-4112 | KJ778656.1 | 99 |
| 35 | *Bacillus* sp. Ob 06 isolate Ob 06 | AJ971891.1 | 70 |
| 36 | *Bacillus* sp. SB3.1 | KU740223.1 | 86 |
| 37 | *Bacillus* sp. SMF5 | AJ868359.1 | 99 |
| 38 | *Bacillus* sp. SW3.2 | KU740234.1 | 99 |
| 39 | *Bacillus stratosphericus* strain IHB B 6832 | KF668462.1 | 99 |
| 40 | *Bacillus stratosphericus* strain IHBB 9411 | KR085786.1 | 99 |
| 41 | *Bacillus subtilis* strain p95_H01 | JQ830651.1 | 74 |
| 42 | *Bacillus subtilis* strain SP3 | KT875349.1 | 80 |
| 43 | *Bacillus subtilis* strain SRI2 | KP271983.1 | 83 |
| 44 | *Bacillus subtilis* strain W1 | KC441816.1 | 81 |
| 45 | *Bacillus thuringiensis* strain Po-5 | JX391979.1 | 76 |
| 46 | *Brevibacillus* sp. XYY-2015 | KR528483.1 | 82 |
| 47 | *Citrobacter murliniae* strain E61 | HQ407238.1 | 78 |
| 48 | *Citrobacter* sp. BRRO1 | KT735246.1 | 84 |
| 49 | *Enterobacter asburiae* strain RCB875 | KT261087.1 | 78 |
| 50 | *Enterobacter* sp. A7 16S | JX081588.1 | 78 |
| 51 | *Enterobacter sp.* enrichment culture clone HSL29 | HM461152.1 | 84 |
| 52 | *Escherichia coli* strain BAB-538 | KF535120.1 | 98 |
| 53 | *Gluconobacter nephelii* strain LMG 26773 | NR_118638.1 | 99 |
| 54 | *Lelliottia amnigena* strain ZB04 | CP015774.1 | 100 |
| 55 | *Lysinibacillus fusiformis* strain BN-4 | JN039176.1 | 80 |
| 56 | *Lysinibacillus* sp. TRS6 | KJ617407.1 | 70 |
| 57 | *Lysinibacillus sphaericus* strain Marseille-P827 | LT223595.1 | 99 |
| 58 | *Paenibacillus* sp. KMSDS2 | JF768723.1 | 82 |
| 59 | *Pantoea agglomerans* | DQ392984.1 | 83 |
| 60 | *Pantoea agglomerans* strain A9 | KC434965.1 | 79 |
| 61 | *Pseudomonas plecoglossicida* strain RD_AZLTR_14 | KU597542.1 | 85 |
| 62 | *Raoultella ornithinolytica* B6 | CP004142.1 | 99 |
| 63 | *Staphylococcus epidermidis* strain SEI | CP009046.1 | 99 |
| 64 | *Staphylococcus saprophyticus* strain PW64 | KT726989.1 | 81 |
| 65 | *Staphylococcus sciuri* | AB212276.1 | 99 |
| 66 | *Staphylococcus* sp. CM2E1 | KM874434.1 | 79 |
| 67 | *Staphylococcus warneri* strain JCR-13 | KU714597.1 | 94 |
| 68 | *Staphylococcus warneri* strain LEH1_5A | JN644590.1 | 81 |
| 69 | *Staphylococcus warneri* strain mammoth-17 | LN998066.1 | 99 |
| 70 | *Staphylococcus warneri* strain MBS022 | KT582294.1 | 79 |
| 71 | Uncultured bacterium clone 218002-244 | JQ940965.1 | 83 |
| 72 | Uncultured *Lactobacillales bacterium* isolate DGGE 6PLAB | GQ911039.1 | 79 |

In order to expedite characterization of single culturable isolates to have a collection of unique, distinct OTUs, direct amplicon sequencing was employed on purified, single-band PCR products of 799F/1492U primers, spanning the V5–V9 hypervariable region of 16S rDNA. Analysis of the resulting electropherograms indicated two groups of sequences: one with clear, sharp and undoubtedly-single peaks for each nucleotide (high quality sequences), and another with background, lower-intensity peaks underneath each nucleotide- read peak (low quality sequences). The former group of amplicons corresponded to the 21 isolates in the Table showing nucleotide identity > 98% to database-retrieved accession numbers; the latter group comprised all remaining amplicons, whose sequences showed 70–94% identities to database-retrieved accession numbers. Ongoing studies in our lab are revealing that such “low-quality” sequences (of single-band PCR amplicons from single isolates) likely correspond to intra-genomic variation of 16S rDNA (to be published elsewhere).
