# Supplementary material for: A 16S rDNA PCR-based theoretical to actual delta approach on culturable mock communities revealed severe losses of diversity information
Source: BMC Microbiol. 2019 Apr 8;19:74. doi: 10.1186/s12866-019-1446-2 (PMC6454784; doi:10.1186/s12866-019-1446-2)
Supplement: Supplementary file 2 — Table S2. Selected bacterial endophytes for in silico restriction analysis of V5–V9 region of 16S rDNA with AluI enzyme. In this table, we present the results from a systematic search for a variety of rRNA gene sequences specifically from endophytic bacteria previously described, which were used for the theoretical in silico MBCs used in this study. For the chosen sequences shown in the Table, we aimed at covering the widest possible spectrum of bacterial species, isolated from all major plant organs. (DOCX 38 kb) [file 12866_2019_1446_MOESM2_ESM.docx]

Supplementary Material

**A 16S rDNA PCR-Based Theoretical to Actual Delta Approach on Culturable Mock Communities Revealed Severe Losses of Diversity Information**

**Hellen Ribeiro Martins dos Santos, Caio Suzart Argolo, Ronaldo Costa Argôlo-Filho*, Leandro Lopes Loguercio.**

** Corresponding author:*

E-mail: ronaldoargolo@yahoo.com.br

Post-graduation Program in Genetics and Molecular Biology (PPG-GBM)

Dept. Biological Sciences (DCB)

State University of Santa Cruz (UESC)

Pav. Jorge Amado,

Rod. BR 415, Km 16, Salobrinho,

Ilhéus-BA, 45662-000

BRAZIL

**1. Supplementary Tables**

**Table S2**. Selected bacterial endophytes for *in silico* restriction analysis of V5–V9 region of 16S rDNA with *Alu*I enzyme

| **plant** | **bacterial species** | **organ** | **access no.** | **reference** |
| --- | --- | --- | --- | --- |
| *Brassica napus* | *Bacillus amyloliquefaciens* | seed | NR_116022.1 | (Granér et al., 2003) |
|  | *Bacillus megaterium* |  | DQ408589.1 |  |
|  | *Pantoea agglomerans* |  | DQ307453.1 |  |
|  | *Pseudomonas fluorescens* |  | AY622220.1 |  |
|  | *Pseudomonas putida* |  | KF831017.1 |  |
|  | *Rahnella aquatilis* |  | KF843720.1 |  |
| *Oryza sativa* L. | *Achromobacter xylosoxidans* | root | AF531768 | (Sun et al., 2008) |
|  | *Acidovorax facilis* |  | AF078765.1 |  |
|  | *Curvibacter gracilis* |  | AB109889 |  |
|  | *Deinococcus indicus* |  | AJ549111 |  |
|  | *Delftia tsuruhatensis* |  | AY302438.1 |  |
|  | *Duganella violaceusniger* |  | AJ871470 |  |
|  | *Herbaspirillum frisingense* |  | AJ238359 |  |
|  | *Sinorhizobium terangae* |  | X68388.2 |  |
|  | *Aurantimonas ureilytica* | leaf | DQ883810 | (Ferrando et al., 2012a) |
|  | *Curtobacterium citreum* |  | AM411064 |  |
|  | *Curtobacterium flaccumfaciens* |  | AY273208 |  |
|  | *Pseudomonas syringae* |  | EU906856 |  |
|  | *Sphingomonas azotifigens* |  | AB217473 |  |
|  | *Sphingomonas yabuuchiae* |  | AB071955 |  |
| *Phragmites australis* | *Azospirillum picis* | root | AM922283 | (Li et al., 2010) |
|  | *Bosea minatitlanensis* |  | AF273081 |  |
|  | *Brevundimonas alba* |  | AJ227785 |  |
|  | *Clostridium hylemonae* |  | AB023972 |  |
|  | *Clostridium tertium 1* |  | Y18174 |  |
|  | *Dechloromonas hortensis* |  | AY277621 |  |
|  | *Desulfomicrobium norvegicum* |  | NR_025407 |  |
|  | *Desulfomonile limimaris* |  | NR_025079 |  |
|  | *Filomicrobium insigne* |  | EF117253 |  |
|  | *Hydrogenophaga bisanensis* |  | EF532793 |  |
|  | *Janthinobacterium lividum* |  | Y08846 |  |
|  | *Kaistia soli* |  | EF592609 |  |
|  | *Pleomorphomonas koreensis* |  | AB127972.1 |  |
|  | *Pleomorphomonas oryzae* |  | AB159680.1 |  |
|  | *Prosthecomicrobium mishustinii* |  | FJ560749 |  |
|  | *Rhizobium daejeonense* |  | AY341343 |  |
|  | *Rhodoplanes elegans* |  | D25311 |  |
|  | *Sinorhizobium chiapanecum* |  | EU286550 |  |
| *Raphanus sativus* L. | *Pseudomonas aeruginosa* | root | EU373426.1 | (Seo et al., 2010) |
|  |  |  |  |  |
|  | *Bacillus licheniformis* | leaf | EU373408.1 |  |
|  | *Bacillus subtilis* |  | EU373407.1 |  |
|  | *Myroides odoratimimus* |  | EU373415.1 |  |
|  | *Sphingobacterium siyangensis* |  | EU373423.1 |  |
|  | *Stenotrophomonas maltophilia* |  | EU373409.1 |  |
| *Sambung nyawa* | *Paenibacillus polymyxa* | leaf | EU373421.1 | (Seo et al., 2010) |
|  | *Burkholderia cepacia* | root | EF602562 | (Mendes et al., 2007) |
| Sugarcane | *Burkholderia cenocepacia* |  | EF602552 |  |
|  | *Pantoea ananatis* | stem | EF602555 |  |
|  | *Pantoea stewartii* |  | EF602554 |  |
| *Trifolium pratense* L | *Agrobacterium rhizogenes* | stem | NR_043398 | (Sturz et al., 1997) |

The search for 16S rDNA sequences from previously characterized endophytes was done by regular literature search and analysis, using regular online-search tools (i.e. “*Google*”, “*Google Scholar*”, and “*PubMed*”) and the keywords “endophyte”, “endophytic bacteria”, “endophytic bacterial diversity". Only those accession files with complete sequence and general taxonomic and descriptive information connected to a published article were used. For the 50 chosen sequences in the Table, we attempted to cover the widest possible spectrum of bacterial species, with the respective endophytes being isolated from all major plant organs.

To compose the ‘theoretical’ data for *Alu*I-restriction profiles, each of these sequences was considered as an Operational Taxonomic Unit (‘OTU’); they were processed to identify the V5–V9 region, and *in silico*-digested with the enzyme, through computational scripts developed for these tasks (available upon request). The resulting *Alu*I-fragments (number and sizes) were recorded for each OTU to compose the so called ‘pre-assembly’ data. This information was then considered as the number of theoretical ‘bands’ that would be produced by each member of a MBC, assembled after this analysis (see Methods for details).
