# Supplementary material for: A 16S rDNA PCR-based theoretical to actual delta approach on culturable mock communities revealed severe losses of diversity information
Source: BMC Microbiol. 2019 Apr 8;19:74. doi: 10.1186/s12866-019-1446-2 (PMC6454784; doi:10.1186/s12866-019-1446-2)
Supplement: Supplementary file 3 — Table S3. Composition of mock bacterial communities based on culturable endophytic isolates from cacao. In this table, one can see all the combinations of individual isolates used to compose the MBCs for the actual (post assembly, prior to PCR) communities, including all number of OTUs assessed (i.e., 5, 10, 15, 20, 25 and 30 OTUs). The information on this table is relevant for a better understanding of data shown in Figs. 3 and 4. (DOCX 88 kb) [file 12866_2019_1446_MOESM3_ESM.docx]

**Table S3**

: Composition of MBCs based on culturable endophytic isolates from cacao.

Obs-1:

The MBCs '1/5

*Bacillus*

-A' and '-B' differed by the composition of

*Bacillus*

isolates

Obs-2:

The numbers of isolates in bold-type in the Table refer to those appearing in the individual

*Alu*

I profiles (Fig. 2)

Obs-3:

The MBC 30.2 had to include three

*Bacillus*

isolates (in red), as the maximum of non-

*Bacillus*

OTUs available were 27

***no. bands***

*= ‘post-assembly’ MBCs (Figs. 3 and 4)*

5-

I

5-

II

5-

III

5-

IV

5-

V

**Random**

**no**

***Bacillus***

**1/5**

***Bacillus-A***

**1/5**

***Bacillus-B***

**only**

***Bacillus***

13

**47**

15

6

**1**

32

**49**

**47**

**48**

**3**

54

60

**51**

**49**

**4**

62

61

59

52

**5**

69

65

**63**

60

**8**

**6**

**2**

**5**

**2**

**6**

10-

I

10-

II

10-

III

10-

IV

10-

V

**Random**

**no**

***Bacillus***

**1/5**

***Bacillus-A***

**1/5**

***Bacillus-B***

**only**

***Bacillus***

3

**47**

15

4

**1**

4

**49**

32

6

**3**

5

52

**47**

**47**

**4**

**41**

58

**51**

**48**

**5**

**48**

59

54

**49**

**7**

**50**

60

56

52

**8**

54

61

59

54

11

62

65

61

59

29

**63**

69

**63**

60

33

68

72

**64**

**63**

34

**7**

**8**

**8**

**6**

**8**

**A 16S rDNA PCR-Based Theoretical to Actual Delta Approach on Culturable Mock Communities Revealed Severe Losses of Diversity Information**

Hellen Ribeiro Martins dos Santos, Caio Suzart Argolo, Ronaldo Costa Argôlo-Filho *, Leandro Lopes Loguercio.

* Corresponding author:

E-mail: ronaldoargolo@yahoo.com.br

Post-graduation Program in Genetics and Molecular Biology (PPG-GBM), Dept. Biological Sciences (DCB), State University of Santa Cruz (UESC)

Rod. BR 415, Km 16, Salobrinho, Ilhéus-BA, 45662-000, BRAZIL

MBCs with

***5***

OTUs

MBCs with

***10***

OTUs
